# Peptides Derived of Kunitz-Type Serine Protease Inhibitor as Potential Vaccine Against Experimental Schistosomiasis

**DOI:** 10.3389/fimmu.2019.02498

**Published:** 2019-11-01

**Authors:** Juan Hernández-Goenaga, Julio López-Abán, Anna V. Protasio, Belén Vicente Santiago, Esther del Olmo, Magnolia Vanegas, Pedro Fernández-Soto, Manuel Alfonso Patarroyo, Antonio Muro

**Affiliations:** ^1^Infectious and Tropical Diseases Group (e-INTRO), IBSAL-CIETUS (Biomedical Research Institute of Salamanca-Research Centre for Tropical Diseases at the University of Salamanca), Faculty of Pharmacy, University of Salamanca, Salamanca, Spain; ^2^Wellcome Sanger Institute, Wellcome Genome Campus, Cambridge, United Kingdom; ^3^Department of Pharmaceutical Chemistry, IBSAL-CIETUS, Faculty of Pharmacy, University of Salamanca, Salamanca, Spain; ^4^Fundación Instituto de Inmunología de Colombia (FIDIC), Bogotá, Colombia; ^5^School of Medicine and Health Sciences, Universidad del Rosario, Bogotá, Colombia

**Keywords:** *Schistosoma mansoni*, helminth vaccines, kunitz-type proteins, synthetic peptide, immunomodulator AA0029, ADAD vaccination system

## Abstract

Schistosomiasis is a significant public health problem in sub-Saharan Africa, China, Southeast Asia, and regions of South and Central America affecting about 189 million people. Kunitz-type serine protease inhibitors have been identified as important players in the interaction of other flatworm parasites with their mammalian hosts. They are involved in host blood coagulation, fibrinolysis, inflammation, and ion channel blocking, all of them critical biological processes, which make them interesting targets to develop a vaccine. Here, we evaluate the protective efficacy of chemically synthesized T- and B-cell peptide epitopes derived from a kunitz protein from *Schistosoma mansoni*. Putative kunitz-type protease inhibitor proteins were identified in the *S. mansoni* genome, and their expression was analyzed by RNA-seq. Gene expression analyses showed that the kunitz protein Smp_147730 (Syn. Smp_311670) was dramatically and significantly up-regulated in schistosomula and adult worms when compared to the invading cercariae. T- and B-cell epitopes were predicted using bioinformatics tools, chemically synthesized, and formulated in the Adjuvant Adaptation (ADAD) vaccination system. BALB/c mice were vaccinated and challenged with *S. mansoni* cercariae. Kunitz peptides were highly protective in vaccinated BALB/c mice showing significant reductions in recovery of adult females (89–91%) and in the numbers of eggs trapped in the livers (77–81%) and guts (57–77%) of mice. Moreover, liver lesions were significantly reduced in vaccinated mice (64–65%) compared to infected control mice. The vaccination regime was well-tolerated with both peptides. We propose the use of these peptides, alone or in combination, as reliable candidates for vaccination against schistosomiasis.

## Introduction

Human schistosomiasis is a water-borne debilitating disease caused by a trematode of the genus *Schistosoma*. It is estimated that 240 million people worldwide are infected with *Schistosoma* spp., which causes the loss of 1.5 million DALYs (Disability Adjusted Life Years) per year (1). In 1994, the WHO (World Health Organization) together with the *Schistosoma* Genome Network started a project aimed to sequencing the *Schistosoma mansoni* genome, which was published in 2009 (2) alongside the *Schistosoma japonicum* genome (3). Three years later, the genome of *Schistosoma haematobium* was described (4). Schistosomes' genome size is relatively large, 409.5 Mbp for *S. mansoni*, 376 Mbp for *S. haematobium*, and 403 Mbp for *S. japonicum* due to the presence of a large number of repetitive sequences (40–45%).

In recent years, high-throughput (next generation) sequencing technologies have provided a large amount of data on covering different aspects of schistosome biology. For example, genome sequencing of multiple isolates has revealed the complex population biology of schistosomes (5, 6), and RNA-seq transcriptomic studies have allowed a better understanding of the gene expression patterns during these parasites' life cycle (7–12). These data are made available to the research community via databases such as GeneDB, SchistoDB, and WormbaseParasite (13–15).

The most interesting schistosome proteins are those related to host–parasite interactions (16), since they are accessible to the effector mechanisms of the host's immune system and may be targets for development of drugs and vaccines against these helminths. There are two promising groups: parasite surface proteins and excretory–secretory proteins. The latter category includes several proteases (serine, cysteine, and aspartic proteases) (17) as well as some protease inhibitors that ensure the survival of the parasite by inhibiting host proteases (18). MEROPS, a database of proteases and inhibitors, contains 1,008 annotated entries for human proteases and homologs (19). The recent availability of the genome sequences of different mammals has allowed the identification of their entire protease composition, termed “degradome,” and its comparison with the human counterpart. The Degradome Database lists 569 human proteases and homologs classified into 68 families (20). A plethora of proteins has been proposed as potential vaccines against schistosomiasis, but only Sm14 and SmTSP-2 vaccines for *S. mansoni* have reached Phase I clinical trials and only the glutathione-S transferase rSh28GST (Bilhvax) against *S. haematobium* has reached Phase III (21).

Kunitz-type protease inhibitors belong to the family of serine protease inhibitors that are found in almost all organisms. They are small proteins containing around 60 amino acid residues (17) and have one or more kunitz motif: α + β with two β strands and two short α helices at the end of the domain. This domain also has three disulfide bonds between six conserved cysteines (22). Kunitz proteins have been involved in various physiological processes such as blood coagulation, fibrinolysis, inflammation, and ion channel blocking (17). However, there is limited information regarding kunitz-type protease inhibitors of parasitic helminths. These molecules have been described in *Fasciola hepatica* (23), *Echinococcus granulosus* (24), and *Ancylostoma* spp. (25) and could be promising antigens for vaccine design. Kunitz-type protease inhibitors have been identified in the genomes of the three major *Schistosoma* spp., but only SjKI-1 from *S. japonicum* and SmKI-1 from *S. mansoni* have been expressed and functionally characterized (26, 27). Recently, recombinant *S. mansoni* kunitz protein (rSmKI-1) formulated with Freund's adjuvant was shown to induce partial protection against C57BL/6 mice infected with *S. mansoni* (28). A strategy to design vaccines is based on the use of conserved peptides involved in critical physiological processes able to interact with major histocompatibility complex (MHC) class I and II molecules and drive protective immune responses. Minimal antigen epitopes with 13- to 18-amino-acid-long peptides can be designed to trigger B- and T-cell immune responses and we can synthesize them chemically (29, 30).

The aim of this study was to explore *S. mansoni* genome *in silico* to identify kunitz-type serine protease inhibitors and to study their expression profile in different life stages by RNA-seq, and to compare them with kunitz protein sequences from other schistosomes and other helminths. One kunitz T-cell and one B-cell epitope were predicted, chemically synthesized, and further tested as potential vaccine candidates against *S. mansoni* in mice.

## Materials and Methods

### Animals and Parasites

Seven-week-old SPF female BALB/c mice (Charles River, Lyon, France) weighing 18–20 g were allocated in standard cages with food and water *ad libitum*, light/dark cycle of 12/12 h, and 22–25°C. Animal procedures complied with the Spanish (L 32/2007, L 6/2013, and RD 53/2013) and the European Union (Di 2010/63/CE) regulations. The Ethics Committee of the University of Salamanca approved animal use protocols (Ref. 15/0018). The size of the groups was calculated by power analysis using the “size.fdr” package in R and following the 3Rs recommendations (31, 32). The animals' health status was monitored during the experiments according to FELASA guidelines. *S. mansoni* was maintained in *Biomphalaria glabrata* snails as intermediate hosts and CD1 mice as definitive hosts. The number of cercariae and their viability were determined using a stereoscopic microscope (Olympus SZX9, Japan).

### Kunitz-Type Protease Inhibitors Study in *S. mansoni* Genome

Amino acid sequences of all putative kunitz domain-containing proteins of *S. mansoni* were retrieved from GeneDB and SchistoDB (14, 15). Sequences containing at least six cysteines were kept for further analyses and aligned with other kunitz proteins from *S. japonicum, S. haematobium, E. granulosus, Echinococcus multilocularis*, and *F. hepatica* available from GeneDB, SchistoDB, GenBank (33), and WormBase ParaSite (13). Amino acid identity between sequences was analyzed using alignments generated with the Clustal Omega online web server (34) and then visually edited with BioEdit software *v*7.1.3 (35). Potential secretory signal peptides were predicted with the SignalP 4.1 online tool (36) with a D-cutoff value of 0.45. Transmembrane helix regions in the sequences were predicted using TMHMM server v2.0 (37). GPI-anchored potential was estimated using fragAnchor (38).

### Kunitz Gene Expression in *S. mansoni* in Cercariae, Schistosomula, and Adults

RNA-seq data from Protasio et al. (7) were used to investigate the expression profile of proteins with kunitz motifs. Fastq files corresponding to samples with accession numbers ERR022873, ERR022874, ERR022876-78, and ERR022880-81 were retrieved from www.ena.ac.uk; reads were mapped to the latest version of the *S. mansoni* genome WBPS12 (https://parasite.wormbase.org/Schistosoma_mansoni_prjea36577/) using HISAT2 v2.1.0 (39) with default parameters except for “–no-mixed –no-discordant.” Output SAM files were converted, sorted, and indexed using SAMTOOLS v1.9 (40). Gene annotation as GFF was obtained from Wormbase ParaSite and corresponds to the database release WBPS12. A GTF version of the annotation was produced using GFFREAD from the CUFFLINKS suite v2.2.1 (41) with options “-F –T.” Counts per gene were computed using FEATURECOUNTS from the SUBREAD v1.6.3 package (42) with default parameters except for “–primary –fraction -t exon -g gene_id.” Counts per gene were further processed using DESeq2 v1.16.1 (43) and visualization of gene expression changes was produced using Integrative Genomics Viewer (IGV) (44) and Tror GGPLOT2 (45) implemented in R (46).

A touchdown PCR (TD-PCR) was developed using the Smp_147730 sequence (Syn. Smp_311670). The reaction was optimized in 25 μl of the reaction mix containing: 2 μl of DNA extracted from *S. mansoni* adults, 13 μl of H_2_O, 2.5 μl of 10 × reaction buffer, 2.5 μl of MgCl_2_ (25 mM), 2.5 μl of dNTPs MIX (25 mM/dNTP), 1 μl (10 pmol) of each primer, and 0.5 μl of Taq-polymerase 2.5 U (iNtRON Biotechnology, Inc). The program consisted of one cycle at 94°C for 1 min, six cycles at 94°C for 20 s, and a touchdown program of 15 cycles with successive annealing temperature decrements from 65 to 60°C for 30 s with 1°C decrement with a final extension at 72°C for 10 min performed in a Mastercycler Gradient (Eppendorf). The products were monitored using 1.5% agarose gel electrophoresis stained with ethidium bromide, visualized under UV light, and then photographed (Gel documentation system, UVItec, UK). The DNA insert obtained was sequenced by the Sanger method at the Sequencing Service of the University of Salamanca.

### B- and T-cell Peptide Prediction and Chemical Synthesis From *S. mansoni* Kunitz Protease Inhibitor

The genetic sequence of the proposed kunitz protease inhibitor gene Smp_147730 (currently Smp_311670 in WBPS12; https://parasite.wormbase.org/Schistosoma_mansoni_prjea36577/) was analyzed *in silico* to identify potential B- and T-cell epitopes that could be soluble and easy to manufacture. Peptide SmKT was designed to induce a good T-cell response using the SYFPEITHI database (47) and the Immune Epitope Database (IEDB) (48); good MHC class II binders were searched for murine H2-E^d^ and human HLA-DRB1. Sequences with scores of more than 20 in predictions based on k-mers of length 15 were selected. The BepiPred server, based on hidden Markov models (HMMs), was used for predicting linear B-cell epitopes (BepiPred 1.0b). Prediction score is based on hydrophilicity and secondary structure prediction (49). The predicted linear B-cell epitope was compared with the results found using the ANTHEPROT 3D software, which takes antigenicity, hydrophilicity, flexibility, and solvent accessibility into account (50). A 20-amino-acid region displaying the best score for each protein was selected as promising linear B-cell epitopes.

The predicted T- and B-cell epitopes (referred to as SmKT and SmKB, respectively) were chemically synthesized at Fundación Instituto de Inmunología de Colombia (FIDIC) (Bogotá, Colombia) by the solid-phase peptide synthesis according to Merrifield (51) and Houghten (52) using the t-Boc strategy and α-benzyhydrylamine (BHA) resin (0.7 meq/mg). One cysteine and a glycine residue were added at both amino and carboxyl-terminal ends to allow their polymerization via oxidization. Peptides were purified by reverse-phase high-performance liquid chromatography, characterized by MALDI-TOF mass spectrometry, and lyophilized. Freeze-dried synthetic peptides were re-suspended in phosphate buffered solution (PBS) and concentrations were determined with a BCA kit of Pierce (Rockford, IL). Peptide toxicity was determined in J774.2 mouse peritoneal macrophage cell line cultures. Cell viability was measured by CytoTox 96^®^ Non-Radioactive Cytotoxicity Assay (Promega) (53).

### Vaccination Trial Using the ADAD Vaccination System

Twenty-one female BALB/c mice were randomly allocated in four groups: Untreated and uninfected group (*n* = 3), Adjuvant treated and infected group (AA0029+Qs) (*n* = 6), SmKT vaccinated and infected group (AA0029+Qs+SmKT) (*n* = 6), and SmKB vaccinated and infected group (AA0029+Qs+SmKB) (*n* = 6). Mice received three vaccinations at 2-week intervals. SmKT and SmKB were formulated in the Adjuvant Adaptation (ADAD) vaccination system with non-hemolytic saponins from *Quillaja saponaria* (Qs; Sigma) and the synthetic aliphatic diamine AA0029 emulsified in a non-mineral oil (Montanide ISA763A, SEPPIC) with a 70/30 oil/water ratio. The ADAD vaccination system is administered using two subcutaneous injections. The first injection contains AA0029 and Qs emulsified in Montanide, and the second injection, administered 5 days after the first, contains the antigen with AA0029 and Qs in the emulsion oil. Individual doses per injection in mice included 100 μg of AA0029, 20 μg of Qs, and 10 μg of either SmKT or SmKB in a final 100-μl volume of emulsion with Montanide based on our prior experience (54, 55). Mice were weighed and monitored for signs of anaphylactic shock, erythema at the injection site, and changes in behavior. Vaccinated and infection control mice were percutaneously challenged with 150 ± 8 *S. mansoni* cercariae by the ring method 2 weeks after the third vaccination (53–55). Mice were restrained with a mixture of 50 mg/kg ketamine (Imalgene1000, Merial), 5 mg/kg diazepam (Valium10, Roche Farma SA), and 1 mg/kg atropine (B. Braun, Madrid) administered intraperitoneally. The abdomen was shaved and wetted with sterile water and then exposed to cercariae for 45 min using a ring (55). All mice were euthanized with a lethal dose of 60 mg/kg of pentobarbital plus 2 IU/ml of heparin and then perfused aseptically with PBS and heparin (500 IU/L). Paired worms as well as single males or females were obtained from the portal and mesenteric veins by portal perfusion at 8 weeks post-challenge. The liver and small intestine were digested in 5% KOH (w/v) overnight at 37°C with shaking, and eggs per gram were counted three times using a McMaster chamber by two different researchers. The spleen, gut, and liver weights were recorded. Liver injury was assessed by the number of granulomas in the surface determined by two pathologists independently using three micrographs (Olympus SZX9) and ImageJ 1.45 software (56).

### Humoral Immune Response by ELISA

Soluble *S. mansoni* adult worm antigens (SoSmAWA) were prepared for ELISA (55). Twenty adult worms per milliliter were suspended in sterile PBS with a protease inhibitor cocktail (Complete Mini EDTA-Free, Roche 04 693 159 001). The mixture was homogenized, frozen and thawed, sonicated, and then centrifuged at 30,000 *g* for 30 min at 4°C. Supernatant protein concentration was determined using a Micro BCA Protein Assay Kit. Blood samples were collected from mice before immunization and infection, and at the necropsy, and analyzed by indirect ELISA to detect specific IgG, IgG1, and IgG2a antibodies anti-SmKT, anti-SmKB, and anti-SoSmAWA. A Corning Costar 96-well microplate (Cambridge, MA) was coated with 1 μg/ml of each peptide and SoSmAWA. The plates were then blocked with 2% of bovine serum albumin (Sigma) in PBS with 0.05% Tween 20 (PBST) for 1 h at 37°C. Sera samples diluted at 1:100 in PBST were added in duplicate wells and incubated for 1 h at 37°C. Goat anti-mouse IgG-HRP, IgG1-HRP, or IgG2a-HRP conjugates (Sigma) were used at 1:1000 in PBST and incubated for 1 h at 37°C. The plates were washed and developed adding H_2_O_2_ (0.012%) and orthophenylenediamine substrate (0.04%) in 0.1 M citrate/phosphate buffer, pH 5.0. The reaction was stopped with 3 N H_2_SO_4_ and read at 492 nm on a MultiSkan GO ELISA plate reader (Thermo Fisher Scientific, Vantaa).

### Parasitological and Immunological Data Analyses

Data were expressed as the mean and standard error of the mean (SEM) and were tested for normality by the Kolmogorov–Smirnov test and homogeneity of variance by the Bartlett test. A one-way ANOVA test and multiple *post-hoc* comparisons with Tukey's honest significance tests (HSD) or Kruskal–Wallis tests were performed to analyze statistical differences among groups. A value of *P* < 0.05 was considered statistically significant. Statistical analyses were performed with SIMFIT Statistical Package 7.4.1 (Manchester University, U. K. https://simfit.org.uk) and SPSS 21 software (IBM).

## Results

While the initial identification of kunitz domain-containing proteins in *S. mansoni* was performed using the former v5.0 of the *S. mansoni* genome assembly (7), an updated and improved version of the assembly was released during the production of this manuscript. Gene accession numbers have changed between these two versions. The original accession numbers (found in v5.0) used to access nucleotide and amino acid sequences in different steps described in the methods of this manuscript were maintained as much as possible to allow cross-referencing with existing literature. However, wherever possible and appropriate, bioinformatics analyses were updated to confirm previous results against the new genome assembly and annotation (WBPS12, https://parasite.wormbase.org/Schistosoma_mansoni_prjea36577/).

### Kunitz-Type Protease Inhibitors Study of *S. mansoni*

The *S. mansoni* genome and gene annotation repositories (GeneDB and SchistoDB) were searched for all kunitz protein sequences. A total of 11 sequences were retrieved with putative kunitz domains in the *S. mansoni* genome. Only three sequences of potential interest remain in V 5.0: Smp_147730, Smp_139840, and Smp_012230. After a close analysis of their amino acid sequence, only Smp_147730 (currently Smp_311670.1) contained a *bona fide* kunitz domain identified between residues 26 and 79, with the six highly conserved cysteine residues capable of establishing three disulfide bonds, found in the range of 50–70 amino acids. In addition, a signal peptide was predicted in the first 21 amino acids of Smp_147730 (Syn. Smp_311670) with a *D* score of 0.855 according to SignalP 4.1 (36). The cleavage site was located between positions 20 and 21 where the *Y* score showed the highest value (*Y* = 0.828). In consequence, residues 1–20 were removed from the B- and T-cell peptide prediction. No transmembrane or GPI anchor domains were found with TMHMM server v2.0 (57).

A new version of the *S. mansoni* genome (version 7, unpublished, available from https://parasite.wormbase.org/Schistosoma_mansoni_prjea36577/Info/Index/, database version WBPS12) was released during the preparation of this manuscript. The gene Smp_147730 has been renamed Smp_311670, and it is predicted to produce two alternative transcripts. The sequence of Smp_311670.1 is identical to our confirmed kunitz protein sequence while Smp_311670.2 represents a longer alternative transcript.

### Comparison of Smp_147730 (Syn. Smp_311670) With Trematode Kunitz Proteins

**Figure 1:**
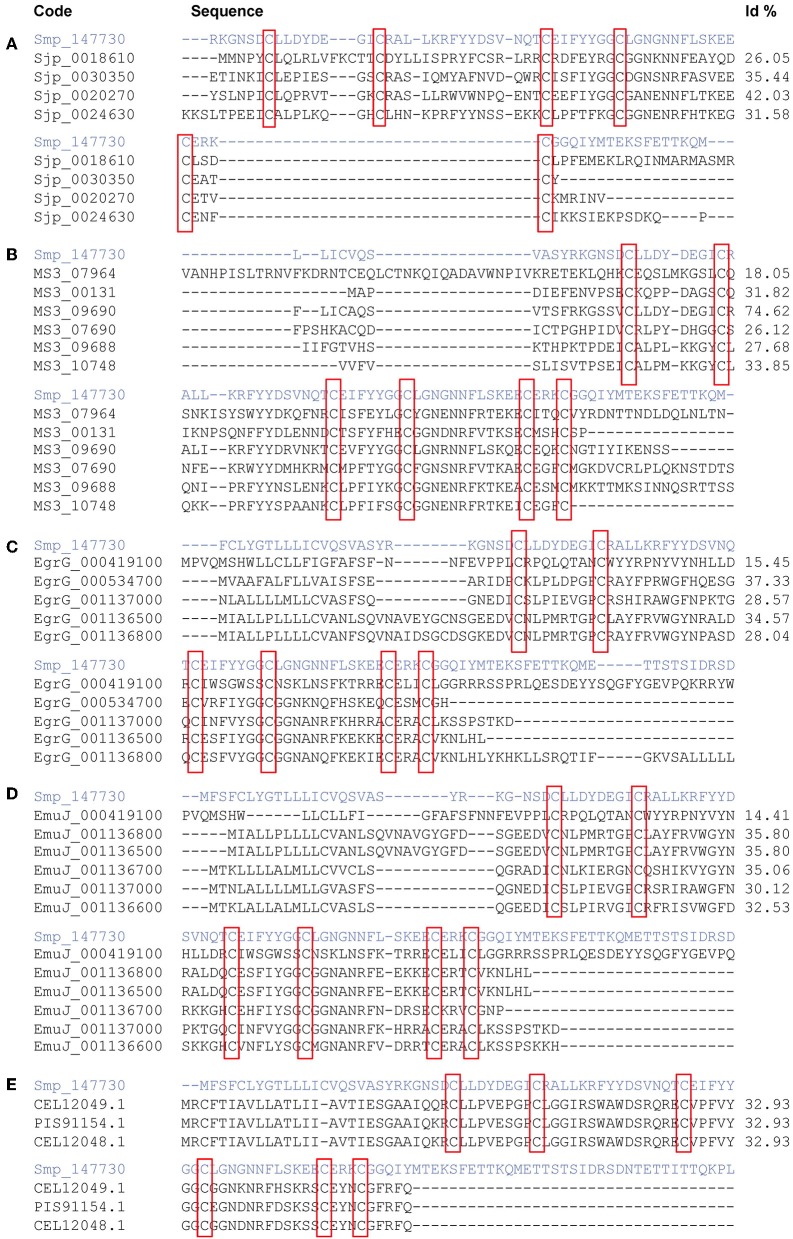
Comparison of sequence Smp_147730 (Syn. Smp_311670) from *Schistosoma mansoni* in a multiple sequence alignment with kunitz-type proteins from *S. japonicum* **(A)**, *S. haematobium* **(B)**, *Echinoccocus granulosus* **(C)**, *E. multilocularis* **(D)**, and *Fasciola hepatica* **(E)**. Kunitz domain positions are highlighted in red boxes.

The Smp_147730 (Syn. Smp_311670) sequence was compared to other putative kunitz proteins of *Platyhelminthes* identified in sequence databases. There were seven sequences retrieved from GeneDB of *S. japonicum* but only four had a six-cysteine kunitz domain with an identity ranging between 26.05 and 42.03% (Figure 1A). Two out of eight *S. haematobium* sequences present in SchistoDB did not include a kunitz domain and the identity of the remaining proteins to Smp_147730 (Syn. Smp_311670) ranged between 18.05 and 74.62% (Figure 1B). Seven kunitz protein sequences in GenBank were attributed to *E. granulosus*, but only five have a kunitz domain and identities ranged from 15.45 to 37.33% (Figure 1C). There were six sequences sharing the domain available in GenBank from *E. multilocularis* with identities from 14.41 to 35.80% out a total of eight retrieved (Figure 1D). Three identical *F. hepatica* sequences were identified in WormBase ParaSite, each presenting a kunitz domain and sharing 32.93% residue identity with Smp_147730 (Figure 1E).

### Transcriptome Analysis and Differential Expression of Smp_147730 (Syn. Smp_311670) Kunitz Gene

**Figure 2:**
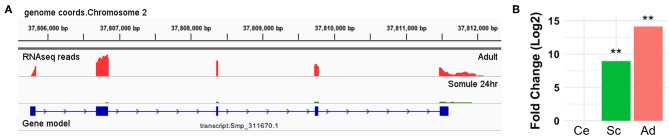
Smp_311670 is up-regulated in intra-mammalian stages. **(A)** Graphical representation using Integrative Genomics Viewer (IGV) of the gene/transcript model Smp_311670 is shown in blue where solid boxes represent exons and the lines and arrows represent introns and the direction of transcription, respectively. The coverage plots above the gene model represent transcriptome (RNA-seq) sequencing reads from two different life cycle stages: adult worms (red) and 24-h schistosomula (green). **(B)** Bar plot showing the relative gene expression levels of Smp_311670 across three life cycle stages, cercariae (fresh water larvae), 24-h schistosomula (intra-mammalian, skin, and migrating), and adult worms (intra-mammalian, mesenteric veins). Values are presented as Log2 fold change relative to cercariae. Ce = cercariae, Sc = 24-h schistosomula, Ad = adult, ^**^Bonferroni adjusted *P* < 0.01.

Smp_311670 is located in Chromosome 2 (37,805,700 and 37,811,500, forward strand) of the WBPS12 *S. mansoni* genome assembly. Transcriptome analysis by RNA-seq showed that Smp_311670.2 was significantly up-regulated (adjusted *p* < 0.01) in 24-h schistosomula and adult worms with respect to cercariae (Figure 2, Supplementary Table 1). No significant difference was found between cercariae and 3-h schistosomula (Supplementary Table 1).

**Figure 3:**
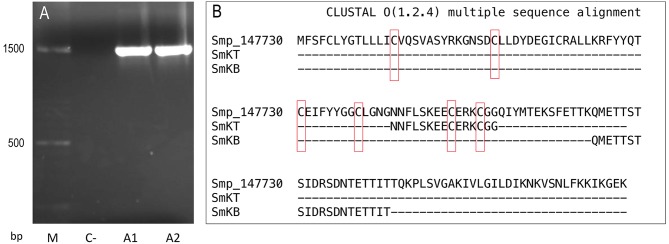
Sequences of Smp-147730 (Syn. Smp_311670) of *S. mansoni*: **(A)** Agarose gel electrophoresis insert of 1444 base pairs (bp) obtained by PCR from two adult DNA samples (A1 and A2) with negative control (C–) and molecular mass marker (M). **(B)** Amino acid sequence and alignment with Clustal Omega of predicted T-cell peptide (SmKT) and B-cell peptide (SmKB).

Primers were designed to amplify Smp_147730 DNA (Syn. Smp_311670) sequence using *S. mansoni* adult DNA, Forw. 5′-TACTGACAGGGCTCACTACGCT-3′ and Rev. 5′-ACGCTCGCCTTCACACCCC-3′ by TD-PCR; the amplified region spanned exons 1 and 2. A 1444-bp insert was obtained and purified by agarose gel electrophoresis, quantified, and sequenced (Figure 3A). A consensus sequence was obtained using Bioedit software and compared through BLAST to sequences recorded in databases.

### T- and B-cell Epitope Prediction and Toxicity Assessment

The online servers SYFPEITHI [http://www.syfpeithi.de (47)] and Immune Epitope Database (IEDB) [http://www.immuneepitope.org/ (48)] were used in the prediction of a 15-amino-acid T-cell peptide (SmKT) in positions 66–80 of Smp_147730 (Syn. Smp_311670) with a score of 22 for H2-Ed and a score of 20 for HLA-DRB1^*^0401 inside the kunitz domain (Figure 3B). A 20-amino-acid B-cell peptide (SmKB) was predicted located on residues 94–114 by BepiPred server [http://www.cbs.dtu.dk/services/BepiPred (47)] and ANTHEPROT [http://antheprot-pbil.ibcp.fr (50)] outside the kunitz domain (Figure 3B). Peptides were obtained with purity more than 90%. Each peptide was assayed ranging from 1 to 50 μg/ml for *in vitro* cytotoxicity evaluation to J774.2 mouse macrophages. Results showed that more than 90% of macrophages were still viable after 3 days of treatment with SmKT and SmKB peptides in all conditions.

### Vaccination With SmKT and SmKB in ADAD With AA0029 Triggers Protection Against *S. mansoni* Infection

**Figure 4:**
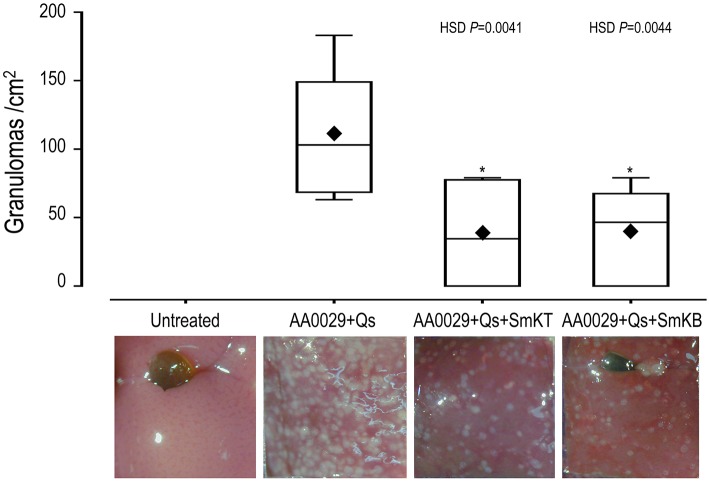
Effect on liver lesion of vaccination with SmKT and SmKB formulated in the Adjuvant Adaptation (ADAD) vaccination system with the synthetic immunomodulator AA0029 and *Quillaja saponaria* saponins (Qs) in BALB/c mice challenged with 150 cercariae of *S. mansoni*. ANOVA *F*_(3, 17)_ = 7.246 and *P* > 0.0024, and *post-hoc* Tukey's honest significance different (HSD) test *P*-values are depicted in the chart. A representative micrograph of each group was included. ♦ represents means. ^*^Significant differences in comparison with AA0029+Qs controls.

The capacity of SmKT and SmKB to induce protection in BALB/c mice against *S. mansoni* infection was evaluated. SmKT, T-cell peptide, formulated in ADAD with the synthetic immunomodulator AA0029 induced higher levels of protection measured by worm recovery with especially high reduction in the number of female worms collected by perfusion (91%; *P* = 0.0002) (Table 1). Also, significant reduction in number of eggs present in the liver (77%; *P* = 0.0044) and in the small intestine (57%; *P* = 0.0208) were detected (Table 1). Liver damage evaluated by the numbers of granulomas on the hepatic surface was also significantly reduced (65%; *P* = 0.0041) compared with controls (Figure 4). BALB/c mice immunized with the SmKB B-cell peptide also showed a high reduction in female worms (89%; *P* = 0.0003) (Table 1), pronounced decreases in the number of eggs present in the liver (81%; *P* = 0.0030) and in the intestine (77%; *P* = 0.0028) (Table 1), as well as a reduced number of granulomas in the liver (64%; *P* = 0.0044) (Figure 4). Both vaccine candidates (SmKT and SmKB) showed comparable protection in terms of eggs trapped in tissues and liver lesions. No signs of anaphylactic shock, erythema, or changes in behavior were observed in vaccinated mice with either peptide. No mice died during the trial. Slight subcutaneous traces of the emulsion were observed at the point of injection.

**Table 1:** Effect of vaccination with SmKT and SmKB formulated in the Adjuvant Adaptation (ADAD) vaccination system on total female and male worms counts, and eggs per gram (EPG) trapped in the tissues of BALB/c mice vaccinated three times at 2-week intervals, challenged 2 weeks after with 150 cercariae of *S. mansoni* and necropsied 8 weeks post-infection.

**Groups**	**Female worms (mean ± SEM)**	***R* (%)**	**Male worms (mean ± SEM)**	***R* (%)**	**EPG in liver (mean ± SEM)**	***R* (%)**	**EPG in gut (mean ± SEM)**	***R* (%)**
AA0029+Qs	11.5 ± 2.8	–	7.7 ± 1.8	–	11,050 ± 2,928	–	11,223 ± 1,609	–
AA0029+Qs+SmKT	1.0 ± 0.4^*^	91	10.7 ± 3.4	NR	2,561 ± 1,510^*^	77	4,872 ± 2,666^*^	57
	*P* = 0.0002				*P* = 0.0044		*P* = 0.0208	
AA0029+Qs+SmKB	1.3 ± 0.4^*^	89	21.7 ± 4.0	NR	2,113 ± 700^*^	81	2,529 ± 933^*^	77
	*P* = 0.0003				*P* = 0.003		*P* = 0.0028	
ANOVA	*F*_(3, 17)_ = 10.721		*F*_(3, 17)_ = 6.787		*F*_(3, 17)_ = 6.243		*F*_(3, 17)_ = 6.112	
	*P* = 0.0003		*P* = 0.0033		*P* = 0.0047		*P* = 0.0052	

*Percentage of reduction (R). Data are presented as the mean and standard error of the mean (SEM). ANOVA and post-hoc Tukey's honest significance test were used*.

* *Significant differences in comparison with AA0029+Qs controls. NR, no reduction*.

### Immunogenicity of *S. mansoni* Kunitz SmKT and SmKB Peptides and Immune Response Against SoSmAWA by ELISA

**Figure 5:**
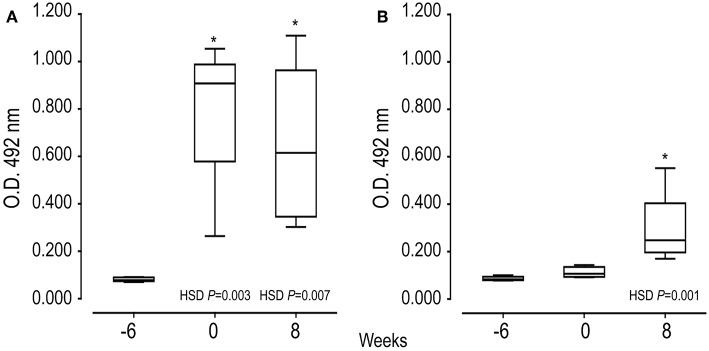
Serum IgG antibody responses of mice vaccinated with SmKB and SmKT by indirect ELISA during vaccination trial: First immunization (−6 weeks), Challenge (0), and Necropsy (8 weeks post-challenge). **(A)** Specific IgG to SmKB of mice vaccinated with AA0029+Qs+SmKB; ANOVA *F*_(2, 15)_ = 10.910, *P* = 0.0017. **(B)** Specific IgG to SmKT of mice vaccinated with AA0029+Qs+SmKT; ANOVA *F*_(2, 15)_ = 10.672, *P* = 0.0013. BABL/c mice were vaccinated using the ADAD vaccination system and then challenged with 150 cercariae of *S. mansoni* and euthanized 8 weeks post-infection. ^*^P < 0.05 compared to serum sample before treatments.

Indirect ELISA tests were performed to examine the ability of SmKT and SmKB to induce humoral immune responses. A significantly higher production of anti-SmKB-specific IgG was observed in mice vaccinated with AA0029+Qs+SmKB compared to the uninfected group after the second immunization (*P* = 0.0030), which was maintained until the end of the experiment (Figure 5A). However, anti-SmKT-specific IgG antibodies were only observed in AA0029+Qs+SmKT vaccinated mice at 8 weeks post-infection (Figure 5B). Nonetheless, antibody response to vaccination with SmKT was lower than that elicited by SmKB (Figure 5).

Specific IgG, IgG1, and IgG2a responses against soluble worm antigen (SoSmAWA) were studied using an indirect ELISA. All infected groups showed an increase in total IgG production to SoSmAWA at 8 weeks post-challenge (0.584 ± 0.118 to 0.720 ± 0.107) compared to the uninfected group (0.250 ± 0.064). All infected groups showed a significant higher production of IgG1 at 8 weeks post-challenge (0.746 ± 0.117 to 0.851 ± 0.107) in comparison with the uninfected group (0.263 ± 0.005) and no significant increase of IgG2a antibodies to SoSmAWA was found during the experiment compared with the uninfected group (Table 2).

**Table 2:** Serum IgG, IgG1, and IgG2a antibody response against soluble adult worm antigen (SoSmAWA) at 8 weeks post-challenge of mice vaccinated with SmKT and SmKB by indirect ELISA.

**Groups**	**IgG (mean ± SEM)**	**IgG1 (mean ± SEM)**	**IgG2a (mean ± SEM)**
Untreated	0.250 ± 0.064	0.263 ± 0.005	0.118 ± 0.027
AA0029+Qs	0.720 ± 0.107^*^	0.851 ± 0.107^*^	0.219 ± 0.036
	*P* = 0.0190	*P* = 0.0134	
AA0029+Qs+SmKT	0.584 ± 0.108^*^	0.746 ± 0.117^*^	0.194 ± 0.029
	*P* = 0.0450	*P* = 0.0476	
AA0029+Qs+SmKB	0.621 ± 0.097^*^	0.804 ± 0.084^*^	0.183 ± 0.039
	*P* = 0.0317	*P* = 0.0239	
ANOVA	*F*_(3, 17)_ = 5.265	*F*_(3, 17)_ = 4.565	*F*_(3, 17)_ = 1.005
	*P* = 0.0210	*P* = 0.0160	*P* = 0.4148

*BALB/c mice were vaccinated using the ADAD vaccination system and then challenged with 150 S. mansoni cercariae and euthanized 8 weeks post-infection. Data are presented as the mean and standard error of the mean (SEM). ANOVA and post-hoc Tukey's honest significance test were used*.

* *Significant differences regarding untreated controls*.

## Discussion

Important progress combating schistosomiasis has been made from 2013 to 2016 as reflected in the reduction of case numbers from 290 to 190 million (1, 58). This decrease in disease burden was mainly achieved through mass preventive chemotherapy with large-scale praziquantel administration complemented with safe water supplies, sanitation, hygiene education, and snail control. There is increasing pressure for the development of new anti-schistosomiasis drugs. Praziquantel, the drug of choice for schistosomiasis, has limitations because it acts only against the adult stage of the schistosome life cycle. In addition, there are major concerns regarding the emergence of drug resistance and/or reduced susceptibility to praziquantel due to its extensive use (59). Meanwhile, the development of a vaccine against this parasite is still high in the research agenda because it would complement the use of praziquantel to reduce disease, stop transmission, and eradicate the disease. Purified or recombinant proteins from schistosomes, host–parasite interface antigens in tegument or gastrodermis, or genome mining by reverse vaccinology have been tested as vaccine candidates but only glutathione-S transferase rSh28GST (Bilhvax) have reached clinical trials (60).

Schistosome kunitz-type serine protease inhibitors have been associated to successful invasion, migration, and development of the parasite in their host. They act by neutralizing the destructive action of host proteases on the invading schistosome (61). In other trematodes, the secretion of proteins with kunitz domains interferes with the maturation of host dendritic cells and regulate host proteases resulting in impairment of defense responses (17). The kunitz protein SmKI-1 isolated from *S. mansoni* was found in excretory–secretory products and tegument of adults as well as eggs. It was observed that it also impairs neutrophil chemotaxis and elastase activity, coagulation, and inflammation mechanisms in the host, inducing immune evasion to ensure their survival. Moreover, the recombinant SmKI-1 delayed blood clot formation, inhibited several trypsin proteases, but had no effect on pancreatic elastase or cathepsins (27). Therefore, kunitz proteins are desirable new targets for vaccine development against schistosomes. Recombinant rSmKI-1 has previously been tested as a vaccine candidate as well as fragments involving the kunitz domain and the C-terminal tail (28, 62).

The potential of kunitz domain-containing proteins as vaccines led us to study the published sequences of these genes in the three main schistosome species (63). We found 11 candidate DNA sequences containing the kunitz domain in several genome annotations of *S. mansoni*, but only Smp_147730 (Syn. Smp_311670) had a six-cysteine residue characteristic of a *bona fide* kunitz domain. Also, we compared Smp_147730 (Syn. Smp_311670) with predicted kunitz-type proteins available in database from *S. haematobium, S. japonicum, E. granulosus, E. multilocularis*, and *F. hepatica*. The identity of the sequence with *S. haematobium* was the highest, up to 74%, but in the other parasites, it was much lower, 43%. This indicates that although the structure of the kunitz domain was preserved in the different species, these proteins could evolve separately and could be species-specific. These sequence differences correspond with the wide functional diversity of kunitz proteins in several species (64).

We focused on Smp_147730 (Syn. Smp_311670), studying its expression by RNA-seq and its identification by PCR in the *S. mansoni* strain maintained in our laboratory. We observed high expression of Smp_147730 (Syn. Smp_311670) after the transformation from cercaria to schistosomulum and even higher expression in the adult stage, suggesting a role in schisosomulum development and the prolonged exposure in portal mesenteric veins of adults. The skin- or lung-migrating schistosomula and adult stages are regarded as major targets to design vaccines against schistosomes (65). With this in mind, our strategy was to design new synthetic high-affinity peptide candidates composed of a short chain of amino acids containing the specific antigen determinant against functional regions that the parasite needs to survive (66). Several epitopes included in a vaccine would trigger humoral and cellular protective response using an adequate adjuvant or delivery system (67). We designed a T-cell peptide of 15 amino acids (SmKT), a candidate from Smp_147730 sequence (Syn. Smp_311670) putatively able to stimulate mouse and human MHC class II, and a linear B-cell peptide of 20 amino acids (SmKB) based on physicochemical properties able to produce a humoral response. These *in silico* analyses are considered feasible, fast, and accurate in designing subunit vaccines against infectious diseases and could produce chemically and structurally defined safer vaccines that are easier to manufacture and store than conventional ones (68). We formulated these two candidates in the ADAD vaccination system developed by our group to overcome the issues of the experimental Freund's adjuvant (54, 55). The ADAD vaccination system has limitations due to the number of injections, needing significant administration improvements to be used in clinical trials.

We next examined whether T- and B-cell epitopes could induce protection in BALB/c mouse experimental schistosomiasis. Both SmKT and SmKB candidates conferred a partial protection in terms of reduction in female worms, eggs trapped in tissues, and liver lesions. These peptides could be useful to reduce liver granuloma pathology, and severe colonic damage and polyps. Fewer eggs in intestines could lead to less passage of eggs in feces and consequently could reduce transmission. The protection is higher than those obtained with the approaches of Morais et al. (28) (34–43%) and Ranasinghe et al. (69) (36–47%) using the whole recombinant rSmKI-1 or with the C-terminal tail fragment (28–30%) (28) using Quil A with a CBA mouse model or Freund's adjuvant and C57BL/6 mice. While these different levels of protection could be explained by differences in adjuvant and animal model, they all indicate the potential of Smp_147730 (Syn. Smp_311670) as a good vaccine candidate. Our peptides seem to act against female worms leading to lower production of eggs and fewer lesions. Curiously, our SmKT peptide of 15-mer including only two cysteines of the conserved kunitz domain induced protection when the KI fragment of 62-mer involving three cysteines conserving the inhibitory activity against trypsin and neutrophil elastase tested by Morais et al. (28) did not.

The immunogenicity was monitored by studying specific IgG against SoSmAWA and both candidates SmKT and SmKB. The early specific IgG response to the 20-mer SmKB peptide observed here was promising; this result and that described against the 67-mer C-terminal tail fragment containing the antiprotease activity described by Morais et al. (28) highlight the role of B-cell-mediated antibody responses in schistosomiasis (62). Numerous literature reports have shown that the high production of specific IgG contributes toward controlling the adult phase of the worm in individuals highly resistant to infection living in hyperendemic areas (70–72) and in experimental models (73). By contrast, the SmKT peptide induced weak antibody responses with only a significant increase at week 8 post-infection, indicating that the protection induced is not solely related with antibody production. No significant variation in the recognition of SoSmAWA in mice vaccinated with either SmKT or SmKB, compared with the infection group, was observed, possibly related to the notion that conserved regions involved in critical biological functions for the parasite are poorly antigenic, but can be modified to render them immunogenic and protection-inducing (30). Further studies aimed at analyzing in depth the cellular immune response induced, particularly by SmKT, are needed, considering that the protective response achieved with this candidate do not seem to solely depend on the antibody levels reached.

## Conclusion

Here, we provide evidence for the protective capacity of two peptides SmKT and SmKB derived from kunitz proteins of *S. mansoni*. These peptides induced reduction in female worms, eggs in tissues, and hepatic damage when administered subcutaneously formulated in the ADAD vaccination system. A single epitope vaccine could be insufficient to trigger a high level of protection, and thus, the combination with other synergic candidates in a multi-antigen vaccine must be tested in order to improve protection against *S. mansoni*.

## Data Availability Statement

The datasets generated for this study can be found in GeneDB SchistoDB. Wormbase ParaSite, Smp_147730, Smp_311670; ERR022873, ERR022874, ERR022876-78, and ERR022880-81.

## Ethics Statement

Animal procedures complied with the Spanish (L 32/2007, L 6/2013, and RD 53/2013) and the European Union (Di 2010/63/CE) regulations. The Ethics Committee of the University of Salamanca approved animal use protocols (Ref. 15/0018).

## Author Contributions

Conception or design of the work: JH-G, JL-A, MP, and AM. Acquisition, analysis, or interpretation of data for the work: JH-G, JL-A, AP, BV, EO, MV, and PF-S. Drafting the work or revising it critically: JH-G, JL-A, AP, MP, and AM.

### Conflict of Interest

The authors declare that the research was conducted in the absence of any commercial or financial relationships that could be construed as a potential conflict of interest.

## References

[1] GBD 2016 Disease and Injury Incidence and Prevalence Collaborators Global, regional, and national incidence, prevalence, and years lived with disability for 328 diseases and injuries for 195 countries, 1990-2016: a systematic analysis for the Global Burden of Disease Study 2016. Lancet. (2017) 390:1211–59. 10.1016/S0140-6736(17)32154-2 28919117 PMC5605509

[2] Berriman M Haas BJ LoVerde PT Wilson RA Dillon GP Cerqueira GC. The genome of the blood fluke *Schistosoma mansoni*. Nature. (2009) 460:352–8. 10.1038/nature08160 19606141 PMC2756445

[3] Zhou Y Zheng H Chen Y Zhang L Wang K Guo J The *Schistosoma japonicum* genome reveals features of host-parasite interplay. *Schistosoma japonicum* Genome Sequencing and Functional Analysis Consortium. Nature. (2009) 460:345–51. 10.1038/nature08140 19606140 PMC3747554

[4] Young ND Jex AR Li B Liu S Yang L Xiong Z. Whole-genome sequence of *Schistosoma haematobium*. Nat Genet. (2012) 44:221–5. 10.1038/ng.1065 22246508

[5] Crellen T Allan F David S Durrant C Huckvale T Holroyd N. Whole genome resequencing of the human parasite *Schistosoma mansoni* reveals population history and effects of selection. Sci Rep. (2016) 6:20954. 10.1038/srep20954 26879532 PMC4754680

[6] Young ND Chan KG Korhonen PK Min Chong T Ee R Mohandas N. Exploring molecular variation in *Schistosoma japonicum* in China. Sci Rep. (2015) 5:17345. 10.1038/srep17345 26621075 PMC4664899

[7] Protasio AV Tsai IJ Babbage A Nichol S Hunt M Aslett MA. A systematically improved high quality genome and transcriptome of the human blood fluke *Schistosoma mansoni*. PLoS Negl Trop Dis. (2012) 6:e1455. 10.1371/journal.pntd.0001455 22253936 PMC3254664

[8] Almeida GT Amaral MS Beckedorff FC Kitajima JP DeMarco R Verjovski-Almeida S. Exploring the *Schistosoma mansoni* adult male transcriptome using RNA-seq. Exp Parasitol. (2012) 132:22–31. 10.1016/j.exppara.2011.06.010 21745473

[9] Piao X Cai P Liu S Hou N Hao L Yang F. Global expression analysis revealed novel gender-specific gene expression features in the blood fluke parasite *Schistosoma japonicum*. PLoS ONE. (2011) 6:e18267. 10.1371/journal.pone.0018267 21494327 PMC3071802

[10] Portela J Grunau C Cosseau C Beltran S Dantec C Parrinello H. Whole-genome in-silico subtractive hybridization (WISH)–using massive sequencing for the identification of unique and repetitive sex-specific sequences: the example of *Schistosoma mansoni*. BMC Genomics. (2010) 11:387. 10.1186/1471-2164-11-387 20565937 PMC3091631

[11] Protasio AV Dunne DW Berriman M. Comparative study of transcriptome profiles of mechanical- and skin-transformed *Schistosoma mansoni* schistosomula. PLoS Negl Trop Dis. (2013) 7:e2091. 10.1371/journal.pntd.0002091 23516644 PMC3597483

[12] Protasio AV van Dongen S Collins J Quintais L Ribeiro DM Sessler F. MiR-277/4989 regulate transcriptional landscape during juvenile to adult transition in the parasitic helminth *Schistosoma mansoni*. PLoS Negl Trop Dis. (2017) 11:e0005559. 10.1371/journal.pntd.0005559 28542189 PMC5459504

[13] Howe KL Bolt BJ Shafie M Kersey P Berriman M. WormBase ParaSite - a comprehensive resource for helminth genomics. Mol Biochem Parasitol. (2017) 215:2–10. 10.1016/j.molbiopara.2016.11.005 27899279 PMC5486357

[14] Zerlotini A Aguiar ER Yu F Xu H Li Y Young ND. SchistoDB: an updated genome resource for the three key schistosomes of humans. Nucleic Acids Res. (2013) 41:D728–31. 10.1093/nar/gks1087 23161692 PMC3531198

[15] Logan-Klumpler FJ De Silva N Boehme U Rogers MB Velarde G McQuillan JA. GeneDB–an annotation database for pathogens. Nucleic Acids Res. (2012) 40:D98–108. 10.1093/nar/gkr1032 22116062 PMC3245030

[16] Braschi S Wilson RA. Proteins exposed at the adult schistosome surface revealed by biotinylation. Mol Cell Proteomics. (2006) 5:347–56. 10.1074/mcp.M500287-MCP200 16269422

[17] Ranasinghe SL McManus DP. Protease inhibitors of parasitic flukes: emerging roles in parasite survival and immune defence. Trends Parasitol. (2017) 33:400–13. 10.1016/j.pt.2016.12.013 28089171

[18] Zhang X Maizels RM Serine proteinase inhibitors from nematodes and the arms race between host and pathogen. Trends Biochem Sci. (2001) 26:191–7. 10.1016/S0968-0004(00)01761-8 11246026

[19] Rawlings ND Barrett AJ Thomas PD Huang X Bateman A Finn RD. The MEROPS database of proteolytic enzymes, their substrates and inhibitors in 2017 and a comparison with peptidases in the PANTHER database. Nucleic Acids Res. (2018) 46:D624–32. 10.1093/nar/gkx1134 29145643 PMC5753285

[20] Pérez-Silva JG Español Y Velasco G Quesada V. The Degradome database: expanding roles of mammalian proteases in life and disease. Nucleic Acids Res. (2016) 44:D351–5. 10.1093/nar/gkv1201 26553809 PMC4702854

[21] Merrifield M Hotez PJ Beaumier CM Gillespie P Strych U Hayward T. Advancing a vaccine to prevent human schistosomiasis. Vaccine. (2016) 34:2988–91. 10.1016/j.vaccine.2016.03.079 27036511

[22] Nixon AE Wood CR. Engineered protein inhibitors of proteases. Curr Opin Drug Discov Dev. (2006) 9:261–8. 16566296

[23] Bozas SE Panaccio M Creaney J Dosen M Parsons JC Vlasuk GV. Characterisation of a novel Kunitz-type molecule from the trematode *Fasciola hepatica*. Mol Biochem Parasitol. (1995) 74:19–29. 10.1016/0166-6851(95)02478-6 8719242

[24] Ranasinghe SL Fischer K Zhang W Gobert GN McManus DP. Cloning and Characterization of Two Potent Kunitz Type Protease Inhibitors from *Echinococcus granulosus*. PLoS Negl Trop Dis. (2015) 9:e0004268. 10.1371/journal.pntd.0004268 26645974 PMC4672886

[25] Hawdon JM Datu B Crowell M. Molecular cloning of a novel multidomain Kunitz-type proteinase inhibitor from the hookworm *Ancylostoma caninum*. J Parasitol. (2003) 89:402–7. 10.1645/0022-3395(2003)089[0402:MCOANM]2.0.CO;2 12760667

[26] Ranasinghe SL Fischer K Gobert GN McManus DP. A novel coagulation inhibitor from *Schistosoma japonicum*. Parasitology. (2015) 142:1663–72. 10.1017/S0031182015001328 26463744

[27] Ranasinghe SL Fischer K Gobert GN McManus DP. Functional expression of a novel Kunitz type protease inhibitor from the human blood fluke *Schistosoma mansoni*. Parasit Vectors. (2015) 8:408. 10.1186/s13071-015-1022-z 26238343 PMC4524284

[28] Morais SB Figueiredo BC Assis NRG Homan J Mambelli FS Bicalho RM. *Schistosoma mansoni* SmKI-1 or Its C-Terminal fragment induces partial protection against *S. mansoni* infection in mice. Front Immunol. (2018) 9:1762. 10.3389/fimmu.2018.01762 30105029 PMC6077287

[29] Stern LJ Calvo-Calle JM. HLA-DR: molecular insights and vaccine design. Curr Pharm Des. (2009) 15:3249–61. 10.2174/138161209789105171 19860674 PMC3615543

[30] Patarroyo ME Patarroyo MA. Emerging rules for subunit-based, multiantigenic, multistage chemically synthesized vaccines. Acc Chem Res. (2008) 41:377–86. 10.1021/ar700120t 18266328

[31] Charan J Kantharia ND. How to calculate sample size in animal studies? J Pharmacol Pharmacother. (2013) 4:303–6. 10.4103/0976-500X.119726 24250214 PMC3826013

[32] Festing MF Altman DG. Guidelines for the design and statistical analysis of experiments using laboratory animals. ILAR J. (2002) 43:244–58. 10.1093/ilar.43.4.244 12391400

[33] Miryala SK Anbarasu A Ramaiah S. Discerning molecular interactions: a comprehensive review on biomolecular interaction databases and network analysis tools. Gene. (2018) 642:84–94. 10.1016/j.gene.2017.11.028 29129810

[34] Sievers F Higgins DG. Clustal Omega, accurate alignment of very large numbers of sequences. Methods Mol Biol. (2014) 1079:105–16. 10.1007/978-1-62703-646-7_6 24170397

[35] Hall TA BioEdit: a user-friendly biological sequence alignment editor and analysis program for Windows 95/98/NT. Nucl Acids Symp Ser. (1999) 41:95–8.

[36] Nielsen H. Predicting secretory proteins with SignalP. Methods Mol Biol. (2017) 1611:59–73. 10.1007/978-1-4939-7015-5_6 28451972

[37] Reddy A Cho J Ling S Reddy V Shlykov M Saier MH. Reliability of nine programs of topological predictions and their application to integral membrane channel and carrier proteins. J Mol Microbiol Biotechnol. (2014) 24:161–90. 10.1159/000363506 24992992 PMC4125430

[38] Poisson G Chauve C Chen X Bergeron A. FragAnchor: a large-scale predictor of glycosylphosphatidylinositol anchors in eukaryote protein sequences by qualitative scoring. Genomics Proteomics Bioinformatics. (2007) 5:121–30. 10.1016/S1672-0229(07)60022-9 17893077 PMC5054108

[39] Kim D Langmead B Salzberg SL. HISAT: a fast spliced aligner with low memory requirements. Nat Methods. (2015) 12:357–60. 10.1038/nmeth.3317 25751142 PMC4655817

[40] Li H Handsaker B Wysoker A Fennell T Ruan J Homer N. The sequence Alignment/Map format and SAMtools. Bioinformatics. (2009) 25:2078–9. 10.1093/bioinformatics/btp352 19505943 PMC2723002

[41] Trapnell C Roberts A Goff L Pertea G Kim D Kelley DR. Differential gene and transcript expression analysis of RNA-seq experiments with TopHat and Cufflinks. Nat Protoc. (2012) 7:562–78. 10.1038/nprot.2012.016 22383036 PMC3334321

[42] Liao Y Smyth GK Shi W. The Subread aligner: fast, accurate and scalable read mapping by seed-and-vote. Nucleic Acids Res. (2013) 41:e108. 10.1093/nar/gkt214 23558742 PMC3664803

[43] Love MI Huber W Anders S. Moderated estimation of fold change and dispersion for RNA-seq data with DESeq2. Genome Biol. (2014) 15:550. 10.1186/s13059-014-0550-8 25516281 PMC4302049

[44] Thorvaldsdóttir H Robinson JT Mesirov JP. Integrative Genomics Viewer (IGV): high-performance genomics data visualization and exploration. Brief Bioinform. (2013) 14:178–92. 10.1093/bib/bbs017 22517427 PMC3603213

[45] Wickham H ggplot2: Elegant Graphics for Data Analysis. New York, NY: Springer-Verlag (2009)

[46] R Core Team. R: A Language and Environment for Statistical Computing. Vienna: R Foundation for Statistical Computing (2017). Available online at: https://www.R-project.org/ [Updated March 11, 2019; cited March, 20, 2019).

[47] Rammensee H Bachmann J Emmerich NP Bachor OA Stevanović S. SYFPEITHI: database for MHC ligands and peptide motifs. Immunogenetics. (1999) 50:213–9. 10.1007/s002510050595 10602881

[48] Fleri W Paul S Dhanda SK Mahajan S Xu X Peters B. The immune epitope database and analysis resource in epitope discovery and synthetic vaccine design. Front Immunol. (2017) 8:278. 10.3389/fimmu.2017.00278 28352270 PMC5348633

[49] Parker JM Guo D Hodges RS New hydrophilicity scale derived from high-performance liquid chromatography peptide retention data: correlation of predicted surface residues with antigenicity and X-ray-derived accessible sites. Biochemistry. (1986) 25:5425–32. 10.1021/bi00367a013 2430611

[50] Deléage G Combet C Blanchet C Geourjon C. ANTHEPROT: an integrated protein sequence analysis software with client/server capabilities. Comput Biol Med. (2001) 31:259–67. 10.1016/S0010-4825(01)00008-7 11334635

[51] Merrifield RB. Solid-phase peptide synthesis. Adv Enzymol Relat Areas Mol Biol. (1969) 32:221–96. 10.1002/9780470122778.ch6 4307033

[52] Houghten RA. General method for the rapid solid-phase synthesis of large numbers of peptides: specificity of antigen-antibody interaction at the level of individual amino acids. Proc Natl Acad Sci USA. (1985) 82:5131–5. 10.1073/pnas.82.15.5131 2410914 PMC390513

[53] Rojas-Caraballo J López-Abán J Pérez del Villar L Vizcaíno C Vicente B Fernández-Soto P. *In vitro* and *in vivo* studies for assessing the immune response and protection-inducing ability conferred by *Fasciola hepatica*-derived synthetic peptides containing B- and T-cell epitopes. PLoS ONE. (2014) 9:e105323. 10.1371/journal.pone.0105323 25122166 PMC4133369

[54] Martínez-Fernández AR Nogal-Ruiz JJ López-Abán J Ramajo V Oleaga A Manga-González Y. Vaccination of mice and sheep with Fh12 FABP from *Fasciola hepatica* using the new adjuvant/immunomodulator system ADAD. Vet Parasitol. (2004) 126:287–98. 10.1016/j.vetpar.2004.07.023 15567592

[55] Vicente B López-Abán J Rojas-Caraballo J del Olmo E Fernández-Soto P Muro A. Protection against *Schistosoma mansoni* infection using a *Fasciola hepatica*-derived fatty acid binding protein from different delivery systems. Parasit Vectors. (2016) 9:216. 10.1186/s13071-016-1500-y 27090442 PMC4836169

[56] Schneider CA Rasband WS Eliceiri KW. NIH Image to ImageJ: 25 years of image analysis. Nat Methods. (2012) 9:671–5. 10.1038/nmeth.2089 22930834 PMC5554542

[57] Krogh A Larsson B von Heijne G Sonnhammer EL. Predicting transmembrane protein topology with a hidden Markov model: application to complete genomes. J Mol Biol. (2001) 305:567–80. 10.1006/jmbi.2000.4315 11152613

[58] Global Burden of Disease Study Group 2013 Global, regional, and national incidence, prevalence, and years lived with disability for 301 acute and chronic diseases and injuries in 188 countries, 1990–2013: a systematic analysis for the Global Burden of Disease Study 2013. Lancet. (2015) 386:743–800. 10.1016/S0140-6736(15)60692-4 26063472 PMC4561509

[59] Mutapi F Maizels R Fenwick A Woolhouse M. Human schistosomiasis in the post mass drug administration era. Lancet Infect Dis. (2017) 17:e42–8. 10.1016/S1473-3099(16)30475-3 27988094 PMC7614913

[60] Alsallaq RA Gurarie D Ndeffo Mbah M Galvani A King C. Quantitative assessment of the impact of partially protective anti-schistosomiasis vaccines. PLoS Negl Trop Dis. (2017) 11:e0005544. 10.1371/journal.pntd.0005544 28410369 PMC5406007

[61] Quezada LA McKerrow JH Schistosome serine protease inhibitors: parasite defense or homeostasis? An Acad Bras Cienc. (2011) 83:663–72. 10.1590/S0001-37652011000200025 21670886 PMC4934610

[62] Morais SB Figueiredo BC Assis NRG Alvarenga DM de Magalhães MTQ Ferreira RS. *Schistosoma mansoni* SmKI-1 serine protease inhibitor binds to elastase and impairs neutrophil function and inflammation. PLoS Pathog. (2018) 14:e1006870. 10.1371/journal.ppat.1006870 29425229 PMC5823468

[63] International Helminth Genomes Consortium Comparative genomics of the major parasitic worms. Nat Genet. (2019) 51:163–174. 10.1038/s41588-018-0262-1 30397333 PMC6349046

[64] Fló M Margenat M Pellizza L Graña M Durán R Báez A. Functional diversity of secreted cestode Kunitz proteins: inhibition of serine peptidases and blockade of cation channels. PLoS Pathog. (2017) 13:e1006169. 10.1371/journal.ppat.1006169 28192542 PMC5325619

[65] McWilliam HE Driguez P Piedrafita D McManus DP Meeusen EN. Discovery of novel *Schistosoma japonicum* antigens using a targeted protein microarray approach. Parasit Vectors. (2014) 7:290. 10.1186/1756-3305-7-290 24964958 PMC4080988

[66] Skwarczynski M Toth I. Peptide-based synthetic vaccines. Chem Sci. (2016) 7:842–54. 10.1039/C5SC03892H 28791117 PMC5529997

[67] Yang H Kim DS. Peptide immunotherapy in vaccine development: from epitope to adjuvant. Adv Protein Chem Struct Biol. (2015) 99:1–14. 10.1016/bs.apcsb.2015.03.001 26067814

[68] Curtidor H Patarroyo ME Patarroyo MA. Recent advances in the development of a chemically synthesised anti-malarial vaccine. Expert Opin Biol Ther. (2015) 15:1567–81. 10.1517/14712598.2015.1075505 26239009

[69] Ranasinghe SL Duke M Harvie M McManus DP. Kunitz-type protease inhibitor as a vaccine candidate against *Schistosomiasis mansoni*. Int J Infect Dis. (2018) 66:26–32. 10.1016/j.ijid.2017.10.024 29128645

[70] Alves CC Araujo N dos Santos VC Couto FB Assis NR Morais SB Sm29, but not Sm22.6 retains its ability to induce a protective immune response in mice previously exposed to a *Schistosoma mansoni* infection. PLoS Negl Trop Dis. (2015) 9:e0003537 10.1371/journal.pntd.0003537 25723525 PMC4344193

[71] McManus DP Loukas A. Current status of vaccines for schistosomiasis. Clin Microbiol Rev. (2008) 21:225–42. 10.1128/CMR.00046-07 18202444 PMC2223839

[72] Melo TT Sena IC Araujo N Fonseca CT. Antibodies are involved in the protective immunity induced in mice by *Schistosoma mansoni* schistosomula tegument (Smteg) immunization. Parasite Immunol. (2014) 36:107–11. 10.1111/pim.12091 24558655

[73] El-Shabasy EA Reda ES Abdeen SH Said AE Ouhtit A. Transmission electron microscopic observations on ultrastructural alterations in *Schistosoma mansoni* adult worms recovered from C57BL/6 mice treated with radiation-attenuated vaccine and/or praziquantel in addition to passive immunization with normal and vaccinated rabbit sera against infection. Parasitol Res. (2015) 114:1563–80. 10.1007/s00436-015-4341-2 25786393

